# Density-dependent effects on the weight of female *Ascaris lumbricoides *infections of humans and its impact on patterns of egg production

**DOI:** 10.1186/1756-3305-2-11

**Published:** 2009-02-10

**Authors:** Martin Walker, Andrew Hall, Roy M Anderson, María-Gloria Basáñez

**Affiliations:** 1 Department of Infectious Disease Epidemiology, Faculty of Medicine, St Mary's Campus, Imperial College London, Norfolk Place, London, W2 1PG, UK; 2 Centre for Public Health Nutrition, School of Integrated Health, University of Westminster, 115 New Cavendish Street, London, W1W 6UW, UK

## Abstract

**Background:**

*Ascaris lumbricoides *exhibits density-dependent egg production, a process which has a marked impact on both the transmission dynamics and the stability of the parasite population. Evidence suggests that the egg production of female *Ascaris *is also associated with the size of the worm. If worm size is mediated by density-dependent processes then the size of female worms may have a causal impact upon patterns of *Ascaris *egg production.

**Results:**

We analyse data collected from a cohort of human hosts, and demonstrate that the per host mean weight (a proxy for size) of female *Ascaris *is dependent on the number of infecting females (worm burden) following a pattern of initial facilitation followed by limitation. Applying a negative binomial (NB) generalized linear model (GLM) and a zero-inflated negative binomial (ZINB) model we confirm that the per host female mean weight is significantly associated with per host egg production. Despite these associations, the mean weight of female *Ascaris *has little causal impact on patterns of density-dependent egg output. The ZINB model is able to account for the disproportionately large number of zero egg counts within the data and is shown to be a consistently better fit than the NB model. The probability of observing a zero egg count is demonstrated as being negatively associated with both female worm burden and female mean weight.

**Conclusion:**

The mean weight of female *Ascaris *is statistically significantly associated with egg output, and follows a consistent pattern of facilitation preceding limitation with increasing female worm burden. Despite these relationships, incorporation of female *Ascaris *mean weight into models of egg output has little effect on patterns of density dependence. The ZINB model is a superior fit to the data than the NB model and provides additional information regarding the mechanisms that result in a zero egg count. The ZINB model is shown to be a useful tool for the analysis of individual-based egg output data.

## Background

Density-dependent population processes can occur at each stage of a parasite's lifecycle [1]. For the gastrointestinal (GI) nematodes these include establishment within the host, development and maturation time, adult survival, and female fecundity [2,3]. Density dependence has important implications for both the stability [2] and transmission dynamics [1,3,4] of helminth populations. Incorporation of these processes into mathematical models as accurately as possible is vital for furthering our understanding of important dynamical behaviour, such as the rate of re-infection following chemotherapeutic intervention and the spread of anthelmintic resistance [3-5].

In *Ascaris lumbricoides *infections of humans, density-dependent egg production has been reported; the *per capita* egg output decreasing with increasing number of worms per host (worm burden) [1,6]. Both the severity of density dependence and the level of egg production exhibit marked geographic variability [7]. This variability has implications for the use of egg counts to estimate the intensity of infection [7], and the applicability of transmission models across geographical locations for decision support in view of recent efforts to integrate the control of neglected tropical diseases.

Density-dependent reductions in worm size may be an important factor in *Ascaris *egg output. A positive correlation between worm size and egg production is commonly described in GI nematodes of ruminants (e.g. [8-13]) as well as in *Ascaris *infections of humans [14,15]. A constraint in worm size at high worm burdens may have a causal impact on patterns of density-dependent egg production. Reductions in worm size at high burdens have been described in both natural [12,16] and experimental [17] systems of directly-transmitted helminths in non-human mammals. There is conflicting evidence on the relationship between size and worm burden in *Ascaris *infections of humans. A number of studies have reported no evidence for density-dependent constraints [18-21] whereas the opposite has been described elsewhere [22].

Worm size and egg production may also interact with the host's immune response. In lambs, acquired immune responses to *Teladorsagia *(= *Ostertagia*) *circumcincta *infections are known to control the size of worms and reduce their egg output [8,23-25]. Similar correlations between the host immune response, worm size and egg output have been described in human hookworm infections [26]. Experimental infections of rats with *Strongyloides ratti *have shown that worms are larger and sometimes more fecund in immune-suppressed rats and smaller and sometimes less fecund in immunized animals when compared to controls [27,28]. Furthermore, density-dependent fecundity effects in this nematode species are known to depend on the host immune response [29,30].

Despite a number of factors potentially influencing the egg production of female *Ascaris*, this important demographic and fitness parameter is ubiquitously described in terms of a single variable; the worm burden. This is largely due to the inherent difficulties in applying suitable statistical models to parasitological data [31,32]. Statistical analyses tend to be complicated by the high degree of variability in the egg output from a single host [33-36], highly overdispersed distributions of worms and egg output across a population of hosts [1,37], and the sensitivity and quantitative reliability of the diagnostic technique [35,36,38]. The estimated concentration of eggs may also be biased by host factors such as the volume of faeces produced (e.g., estimates in children tend to be inflated relative to those in adults) [35]. Typically, density-dependent reductions in egg output are presented in terms of female worm fecundity, a composite parameter describing the *per capita *egg production per unit time (eggs per gram of faeces divided by the number of (female) worms per host). Detection of density dependence has frequently been performed by fitting a functional form to the relationship between fecundity and worm burden. This method contravenes assumptions of statistical independence and may introduce bias via inaccuracies in the estimation of worm burden [39].

A number of studies have characterised density-dependent patterns of *Ascaris *egg output by fitting statistical models to grouped mean egg output data (e.g. [7,19,34]). The advantage of this method is that, assuming a large enough sample size per group, the distribution of means can be assumed to be normal, evoking the central limit theorem. However, this complicates the investigation of other variables which may also be important determinants of egg production. In addition, density-dependent *Ascaris *egg output has been exclusively described using data collected from populations at temporal equilibrium; consequently, whether this phenomenon is static or temporally dynamic is not known.

The analyses in this study are split into two parts. In the first part we define and fit a statistical model to evaluate the evidence for different forms of density dependence in the per host mean weight (a proxy for size) of female *Ascaris*. In the second, we explore the relationship between the mean weight of female worms and per host egg output using a multivariate modelling approach (controlling for both female worm burden and host age). We explore the suitability of two types of statistical model for modelling these individual-based egg output data: a negative binomial (NB) generalized linear model (GLM) [40] and a zero-inflated negative binomial (ZINB) model [41,42]. The latter is useful in modelling data with a high proportion of zero counts [42-44] and has been applied to GI nematode egg count data in two previous studies [45,46]. Throughout this paper we define the net egg output as the estimated concentration of eggs per gram of faeces per host (regardless of whether they are or not fertilised). Thus we distinguish between egg production (fecundity) and fertility, whereby the latter measures the number of fertilised and embryonated eggs that a female worm produces (i.e. live offspring).

## Methods

### Study area and data collection

Data were collected from a poor urban suburb of Dhaka, Bangladesh between 1988 and 1989 by Hall and colleagues [7,47,48]. Briefly, households were visited and all their occupants invited to take part in the study with the aim of recruiting as many individuals as possible. All participants were asked to provide a faecal sample from which the number of *Ascaris lumbricoides *eggs were counted using a quantitative ether sedimentation technique [36] and the concentration of eggs per gram of faeces (EPG) estimated. Pyrantel pamoate was administered to each subject and their stools were collected for a period of 48 hours post-treatment. The worms recovered (*A. lumbricoides*) from the faeces of each individual were sexed, counted and weighed. Egg counts, treatment and worm counts were repeated on two further occasions at six-monthly intervals. Pyrantel pamoate paralyses adult *Ascaris *allowing them to be expelled intact from the gut [49] with a cure rate of approximately 88% [50]. Hence, these data provide a reliable and accurate measure of the number and weight of worms per host. The population of worms recovered after the first round of chemotherapy is termed the baseline population, after the 2^nd ^round of chemotherapy, the 1^st ^re-infection population and after the 3^rd ^and final round, the 2^nd ^re-infection population. The pre-treatment egg counts are similarly referred to.

### Sample size

To evaluate the evidence for different forms of density dependence affecting the per host mean weight of female *Ascaris*, analyses were performed on the data collected from all individuals who were found to be infected with at least one female worm. To explore the relationship between the per host mean weight of female *Ascaris *and the per host egg output, data were analysed from those individuals who were found to be infected with at least one female worm and from whom an estimate of egg output had been made. Table 1 summarises the data (available upon request to authors) used in these analyses. Definitions and descriptions of all parameters and variables used throughout this paper are given in Table 2.

**Table 1:** Summary of data used in analyses

Population	Hosts sampled		Hosts lost to follow-up		Hosts with a positive female worm count		Hosts with a positive female worm count and from whom egg output was estimated	
	age ≤ 12	age > 12	age ≤ 12	age > 12	age ≤ 12	age > 12	age ≤ 12	age > 12
	
Baseline	990	775	-	-	834	639	834	639
1^st ^re-infection	699	558	291	217	555	383	528	364
2^nd ^re-infection	592	425	107	133	496	257	463	228

**Table 2:** Definitions of variables and parameters

Type	Symbol	Definition/description	Units
Random variables, observed values	Λ, *λ*	Per host net egg output	eggs gram^-1^
	*W*, *w*	Per host mean weight of female *Ascaris*	grams
	*n*	Per host female worm burden	worms host^-1^
	*a*	Host age group (*a *= 0 for age ≤ 12, *a *= 1 for age > 12,)	

Unobserved random variables	*M*	Per host unobserved true mean weight of female *Ascaris*	grams

Unobserved values	*μ*_*W*_	Expected value of the per host mean weight of female *Ascaris*	grams
	*μ*_Λ_	Expected value of the per host net egg output	eggs gram^-1^
	*p*	The probability of observing a zero count from the Bernoulli process	-

Estimated parameters	$\alpha_{a}=\{\alpha_{1a},\alpha_{2a},\alpha_{3a}\}$	Describe the form of density-dependence of the expected value of the per host mean weight of female *Ascaris *in age group *a*	-
	$\sigma =\{\sigma_{1}^{2},\sigma_{2}^{2}\}$	Describe the relationship between the variance of the per host mean weight of female *Ascaris *and the female worm burden	-
	*λ*_1_	Exponential of the intercept term of statistical models fitting the expected value of the per host net egg output to data	-
	*β*	Describes the effect of host age category on the expected value of the per host net egg output	-
	*c*	An inverse measure of the severity of density dependence (0 <*c *≤ 1) on the expected value of the per host net egg output	-
	$\gamma =\{\gamma_{1},\gamma_{2},\gamma_{3}\}$	Describe the form of the relationship between the expected value of the per host net egg output and female *Ascaris *mean weight	-
	$\delta =\{\delta_{0},\delta_{1},\delta_{2},\delta_{3}\}$	Describe the age-dependent relationship between the logit of the probability of observing a zero egg count, the natural logarithm of female worm burden and the natural logarithm of the mean weight of female *Ascaris*	-
	*k*	Inverse measure of the degree of overdispersion in egg output data	-

### Per host female mean weight and worm burden

To explore the relationship between the mean weight of female *Ascaris *in each host infra-population and the female worm burden we define the following statistical model. Let *n *be the number of female worms in a single host. Given a host harbours *n *female worms, the weights of the individual female worms are assumed to be independent with true mean *M *and variance $\sigma_{1}^{2}$. We further assume that *M *is a random variable with mean $\mu_{W}$ and variance $\sigma_{2}^{2}$. The observed per host average weight of a female worm, *W*, has mean $\mu_{W}$, variance $\sigma_{1}^{2}/n+\sigma_{2}^{2}$ and, for reasonably large *n*, will approximately follow a normal distribution,

(1) $$W\sim N(\mu_{W},\sigma_{1}^{2}/n+\sigma_{2}^{2}).$$

To model different types of density dependence we allow $\mu_{W}$ to be a function of the female worm burden, *n*, using the following generalised equation,

(2) $$\mu_{W}=\frac{\alpha_{1}n^{\left(\alpha_{3}-1\right)}}{\left(1+\alpha_{2}n^{\alpha_{3}}\right)}.$$

This function can model three different possible relationships between $\mu_{W}$ and *n*. Model A: $\mu_{W}$ can be a positive constant for all $n(\alpha_{1}>0,\alpha_{2}=0,\alpha_{3}=1)$, indicative of an absence of density-dependent effects. Model B: $\mu_{W}$ can decline asymptotically with increasing $n(\alpha_{1}>0,\alpha_{2}>0,\alpha_{3}=1)$, representing negative or constraining density dependence. Model C: $\mu_{W}$ can initially increase with increasing *n *followed by an asymptotic decline $\left(\alpha_{1}>0,\alpha_{2}>0,\alpha_{3}>1\right)$ describing a pattern of initial facilitation (positive density dependence) followed by limitation (negative density dependence); for examples and further discussion of using forms of equation (2) to describe density dependence in other host-parasite systems see [51,52]. Each of Models A-C is nested within the following one allowing their respective fits to be compared using the likelihood-ratio statistic (LRS) [53]. Under the null hypothesis the LRS follows a chi-square distribution with degrees of freedom (d.f.) equal to the difference in the number of parameters being estimated [54]. Akaike's information criterion (AIC) [55] was also calculated as an additional measure of goodness-of-fit. Host age, *a*, was incorporated into the model as a two level factor (*a *= 0 for children ≤12 years, *a *= 1 for teenagers and adults > 12 years) to allow the parameters pertaining to density dependence, $\alpha_{a}=\{\alpha_{1a},\alpha_{2a},\alpha_{3a}\}$, to vary between age groups. The variance parameters, $\sigma =\{\sigma_{1}^{2},\sigma_{2}^{2}\}$, were considered independent of host age. Assuming the observed per host mean female weight data to follow a normal distribution of the form given in equation (1), a log-likelihood function was derived and maximised using the quasi-Newton Broyden-Fletcher-Goldfarb-Shanno (BFGS) [56-59] optimisation algorithm to obtain maximum likelihood estimates (MLEs) of the unknown parameters (***α***_*a *_and ***σ***). The BFGS algorithm was implemented using the optim function in the R statistical program (v.2.8.0) [60,61]. The best-fit form of equation (2) was determined separately in each of the baseline, 1^st ^and 2^nd ^re-infection populations.

### Per host female mean weight and net egg output

#### Model derivation

For *A. lumbricoides*, the relationship between the total net egg output per host (denoted by the random variable *Λ*) and the per host female worm burden, *n*, has been empirically well described by a power function [7],

(3) $$E(\Lambda )=\mu_{\Lambda }=\lambda_{1}n^{c}.$$

Here *E *represents the expected value, *λ*_1 _the number of eggs per gram of faeces produced by a sole infecting female and and *c *inverse measure of the severity of negative density dependence (for 0 <*c *≤ 1), with *c *= 1 indicating proportionality or density independence. This model is conveniently linearised by taking natural logarithms,

(4) $$\mathrm{Ln}(\mu_{\Lambda })=\mathrm{Ln}(\lambda_{1})+c\mathrm{Ln}(n).$$

A null model was defined by extending this relationship to include host age, *a *(where *c *is once again a two-level factor), by adding a multiplicative factor, *e*^*β *^[in equation (3)], for teenagers and adults ≤12 years. Host age is a potentially important confounding factor associated with both the number of worms per host (the age-intensity profile of *Ascaris *infection is typically convex, for examples see [6,48]), and the concentration of egg counts (egg counts tend to be negatively related with the volume of faeces produced resulting in overestimation in children compared to adults [35]). Thus, the null model (denoted Model 1) describing the relationship between net egg output and female worm burden adjusting for host age was defined as,

(5) $$\mathrm{Ln}(\mu_{\Lambda })=\mathrm{Ln}(\lambda_{1})+c\mathrm{Ln}(n)+\beta a.$$

**Figure 1:**
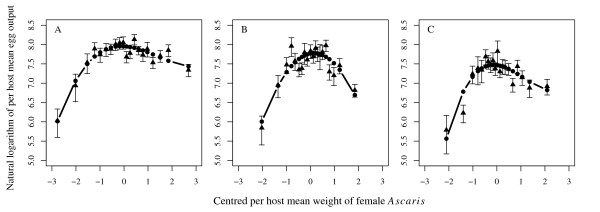
**The relationship between the per host net egg output and mean weight of female *Ascaris lumbricoides***. The relationship between the per host net egg output and the (centred, see main text) mean weight of female *Ascaris *in the baseline (A), 1^st ^(B) and 2^nd ^(C) re-infection populations. Triangles represent grouped mean egg outputs stratified by female *Ascaris *mean weight. Solid lines and circles represent the fitted values of the best fit polynomial functions (as determined by the likelihood-ratio statistic (LRS), see Table 3).

In order to extend Model 1 [equation (5)] to reflect the potential dependence of per host net egg output on per host female mean weight the following preliminary analyses were performed to determine appropriate functional forms to describe the relationship. Per host egg output data were stratified by per host mean weight, taking the arithmetic mean per stratum, and regressing these values against polynomial functions (up to 3^rd ^order) of the mean of the per host mean weight of each stratum. Stratum means were centred around their overall arithmetic mean value in order to minimise multicollinearity [62]. A problem of collinearity for non-centred polynomial terms was indicated by high values (consistently greater than 10 [63]) of their variance inflation factors (VIFs) [64] and high standard errors of their estimated coefficients. (We centre the per host female mean weight in all subsequently described polynomial regression models to ensure robust parameter estimation. We do not continue to explicitly indicate this to maintain the clarity of the mathematical notation.) Models were fitted using standard GLM procedures assuming the mean egg output per stratum to be normally distributed with constant variance [40] and implemented using the glm function in R [60,61]. Models were compared using the LRS and AIC. In the baseline, 1^st ^and 2^nd ^re-infection populations, the 3^rd ^order (cubic), 2^nd ^order (quadratic) and 3^rd ^order functions were, respectively, indicated by both test statistics as being the best fits (Figure 1, Table 3).

**Table 3:** Models describing the egg output of *Ascaris lumbricoides *as polynomials of the mean weight of female worms

Population	Model order polynomial	Maximum log-likelihood	LRS	*p*-value	AIC
Baseline	1^st^	-10.68	-	-	27.37
	2^nd^	4.71	30.80	<0.0001	-1.43
	3^rd†^	11.22	13.02	0.00031	-12.45

1^st ^re-infection	1^st^	-12.98	-	-	31.97
	2^nd†^	2.96	31.88	<0.0001	2.09
	3^rd^	3.88	1.84	0.17	2.25

2^nd ^re-infection	1^st^	-12.14	-	-	30.29
	2^nd^	0.0043	24.30	<0.0001	7.99
	3^rd†^	3.68	7.34	0.0067	2.65

Comparison of models describing the per host net egg output of *Ascaris *as polynomial functions of the per host mean weight of female worms. Analyses were performed on grouped mean data (see text) using standard GLM procedures. ^† ^denotes the best-fit model in each population.

Using the 3^rd ^order relationship, Model 1 [equation (5)] was extended to define a full model describing the relationship between net egg output, female worm burden, and worm weight adjusting for host age,

(6) $$\mathrm{Ln}(\mu_{\Lambda })=\mathrm{Ln}(\lambda_{1})+c\mathrm{Ln}(n)+\beta a+\gamma_{1}w+\gamma_{2}w^{2}+\gamma_{3}w^{3}$$

Equation (6) is denoted Model 4. Models 2 and 3 are defined as special cases of Model 4, modelling female *Ascaris *mean weight as, respectively, 1^st ^order $\left(\gamma_{1}>0,\gamma_{2}=0,\gamma_{3}=0\right)$ and 2^nd ^order $\left(\gamma_{1}>0,\gamma_{2}>0,\gamma_{3}=0\right)$ polynomial functions (Table 4). In these models the parameters pertaining to the mean weight of female *Ascaris *per host $\left(\gamma =\{\gamma_{1},\gamma_{2},\gamma_{3}\}\right)$ do not have a direct biological interpretation although from the unadjusted models (i.e. unadjusted for the effects of host age and female worm burden) in each population (Figure 1), it is clear that the mean egg output tends to initially rise with increasing female mean weight followed by a decline. This functional relationship may be thought of as empirically modelling the antagonistic effects of growth and ageing on worm egg production.

**Table 4:** Equations for Models 1–4 and 1I-4I

Models	Negative binomial count component	Bernoulli component (zero-inflated "I" models only)
1,1I 2,2I 3,3I 4,4I	$\text{Ln}(\mu_{\Lambda })=\text{Ln}(\lambda_{1})+c\text{Ln}(n)+\beta a$ $\text{Ln}(\mu_{\Lambda })=\text{Ln}(\lambda_{1})+c\text{Ln}(n)+\beta a+\gamma_{1}w$ $\text{Ln}(\mu_{\Lambda })=\text{Ln}(\lambda_{1})+c\text{Ln}(n)+\beta a+\gamma_{1}w+\gamma_{2}w^{2}$ $\text{Ln}(\mu_{\Lambda })=\text{Ln}(\lambda_{1})+c\text{Ln}(n)+\beta a+\gamma_{1}w+\gamma_{2}w^{2}+\gamma_{3}w^{3}$	$Logit(p)=\delta_{0}+\delta_{1}a+\delta_{2}\text{Ln}(n)+\delta_{3}\text{Ln}(w)$

### Statistical modelling approach

**Figure 2:**
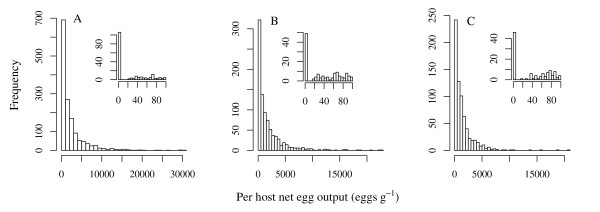
**The distribution of per host egg output**. Histograms depicting the distribution of the per host egg output in the baseline (A), 1^st ^(B) and 2^nd ^(C) re-infection populations. The insets are histograms of the distribution between 0–100 eggs gram^-1 ^highlighting the high proportion of zero counts.

In order to fit these linear models to the data it is necessary to assume an appropriate probability distribution of the per host net egg output, *Λ*. Count data are typically modelled assuming either a Poisson or negative binomial distribution (NBD) [40,42]. Given the high level of overdispersion in the egg output data (variance-to-mean ratio (VMR) = 4855, 4081 and 2726 in the baseline, 1^st ^re-infection and 2^nd ^re-infection populations respectively; see also Figure 2), the NBD is more appropriate than the Poisson [42]. However, these data also comprise a high proportion of zero counts which may not be adequately captured by the NBD (zero inflation, Figure 2). For *A. lumbricoides*, a zero egg count represents either an infra-population containing no sexually mature females or a false negative [38], since even unfertilised females can produced (unfertilised) eggs. (In this study, fertilised eggs were not distinguished from those unfertilised.) The distribution of *Ascaris *eggs in faecal samples from infected individuals has been shown to be highly aggregated [65] making false negatives more likely. Furthermore, the probability of a false negative may be dependent on properties of the worm infra-population and the infected host. Data that are zero inflated relative to the NBD may be better described by a two-component mixture model which defines the response variable as a mixture of a Bernoulli and NBD (zero-inflated negative binomial, ZINB) [41-44,66]. Such a distribution allows zero counts to arise from two distinct mechanisms: a Bernoulli (binary) process generating either a positive or zero count and a count process (including the possibility of a zero count) [42]. Covariates of each process may or may not be the same [44] affording flexibility to construct models with the potential to explain a much higher degree of variability than assuming a single distribution. In these analyses, we fit the linear models derived in the previous section using both a negative binomial and mixture model approach and compare their respective fits.

#### Negative binomial (NB) model

For the NBD the probability of observing an egg count *λ *is,

(7) $$P(\Lambda =\lambda )=\frac{\Gamma (\lambda +k)}{\Gamma (k)\lambda !}\frac{\mu_{\Lambda }k^{k}}{{\left(\mu_{\Lambda }+k\right)}^{\lambda +k}}$$

where *k *is an inverse measure of the degree of overdispersion [61] and Γ is the gamma function [Γ(*x*) = (*x *- 1)!]. For a known value of *k*, equation (7) is of the form of the exponential family of probability distributions [40,67]. The natural logarithm (the link function) of *μ*_Λ_ is linearly related to the covariates described in the previous section [equations (5) and (6)] and so, for a given value of *k*, the parameters can be estimated within the GLM framework [40]. Here, since *k *is unknown, we employ a frequently used extension of the GLM methodology which allows maximum likelihood estimates of both *k *and the unknown linear parameters to be obtained [61,68]. The glm.nb function from the MASS package in R [61] was used to implement this technique and fit Models 1–4 (Table 4) to the data in each population. Models were compared using the LRS and AIC to determine the best-fit. We refer to these models as negative binomial or NB models.

#### Zero-inflated negative binomial (ZINB) model

For the zero-inflated negative binomial distribution the probability of observing an egg count *λ *is,

(8) $$\begin{array}{c} P(\Lambda =0)=p+(1-p){\left[\frac{k}{\left(\mu_{\Lambda }+k\right)}\right]}^{k}\\ \begin{array}{cc} p(\Lambda =\lambda )=(1-p)\frac{\Gamma (\lambda +k)}{\Gamma (k)\lambda !}\frac{\mu_{\Lambda }^{\lambda }k^{k}}{{\left(\mu_{\Lambda }+k\right)}^{\lambda +k}} & \lambda =1,2,... \end{array} \end{array}$$

**Figure 3:**
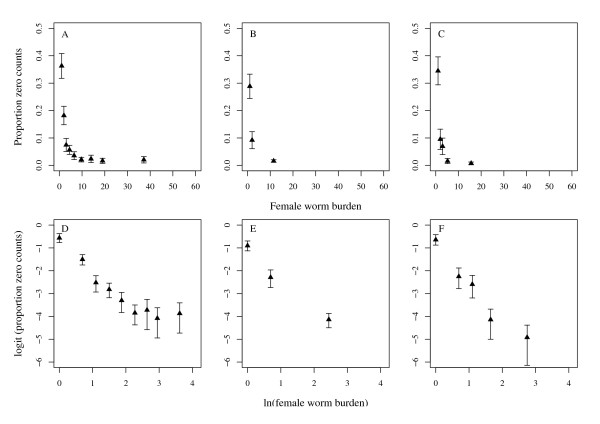
**Relationship between the proportion of zero egg counts and female worm burden**. Top row: a scatter plot of the proportion of zero egg counts per stratum of female *Ascaris *worm burden in the baseline (A), 1^st ^(B) and 2^nd ^(C) re-infection populations. Bottom row: logit of the proportion of zero egg counts per stratum of the natural logarithm of female worm burden in the baseline (D), 1^st ^(E) and 2^nd ^(F) re-infection populations showing approximately linear relationships.

**Figure 4:**
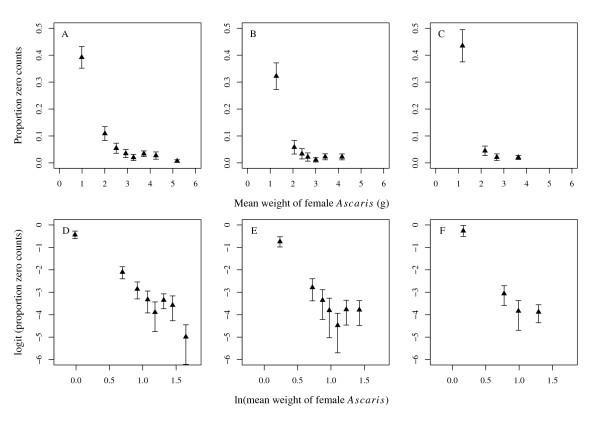
**Relationship between the proportion of zero egg counts and the mean weight of female *Ascaris lumbricoides***. Top row: a scatter plot of the proportion of zero egg counts per stratum of female *Ascaris *mean weight in the baseline (A), 1^st ^(B) and 2^nd ^(C) re-infection populations. Bottom row: logit of the proportion of zero egg counts per stratum of the natural logarithm of female mean weight baseline (D), 1^st ^(E) and 2^nd ^(F) re-infection populations showing approximately linear relationships.

Here *p *is the probability of observing a zero count originating from the Bernoulli process and [*k*/(*μ*_Λ _+ *k*)]^*k *^is the probability of observing a zero count from the NBD. Just as *μ*_Λ _is linearly related to covariates via the logarithmic link function, the logit function (ln[*p*/(1 - *p*)]) can be used to linearise the relationship between *p *and potential covariates [40]. Univariate exploration of the data indicated a negative linear relationship between logit(*p*) and the natural logarithm of stratified groups of the per host mean weight of female *Ascaris *and the per host female worm burden (Figures 3 and 4). Equation (9) is a model which includes host age (*a*), the natural logarithm of the mean weight of female *Ascaris *[ln(*w*)], and the natural logarithm of the female worm burden [ln(*n*)] as covariates of the probability of observing a zero count. This model was fitted to the data in each population using standard GLM procedures implemented in R using the glm function [40,61].

(9) $$\log it(p)=\delta_{0}+\delta_{1}a+\delta_{2}\mathrm{Ln}(n)+\delta_{3}\mathrm{Ln}(w)$$

These preliminary multivariate analyses confirmed ln(*n*) and ln(*w*) to be statistically significantly and negatively related to the probability of observing a zero count in each population (Table 5). In the 2^nd ^re-infection population age was found to be positively associated with *p *(i.e. the probability of observing a zero count is greater in teenagers and adults than in children) (Table 5). The relationship given in equation (9) was used to define the Bernoulli component of the mixture model and extend Models 1–4 into zero-inflated models (denoted by the letter I, Models 1I-4I, Table 4). These models were fitted to the data in each population using maximum likelihood implemented using the zeroinfl function from the pscl package (v.1.02) [68] in R (for further information on fitting zero-inflated mixture models see [41,43,69]). The fitted models were compared to one another using the LRS to determine the best-fit and also to their corresponding NB model (Models 1–4) using AIC. We refer to zero-inflated models as ZINB.

**Table 5:** Parameter values estimated from the logistic model describing the probability of a zero egg count

Population	*δ*_0_ (SE)	*δ*_1_ (SE)	*δ*_2_ (SE)	*δ*_3_ (SE)
Baseline	0.49* (0.24)	-0.23 (0.27)	-1.77*** (0.18)	-1.01*** (0.14)
1^st ^re-infection	0.91* (0.43)	0.51 (0.40)	-2.63*** (0.36)	-2.00*** (0.33)
2^nd ^re-infection	0.63 (0.41)	1.07** (0.41)	-2.56*** (0.38)	-1.62*** (0.28)

Parameter values were estimated by fitting equation (9) to the egg count data encoded in a binary fashion (positive or zero) using standard GLM procedures. Parameters refer to the following covariates: *δ*_1_: host age, *δ*_2_: the natural logarithm of female worm burden and *δ*_3_: the natural logarithm of female mean weight. Parameters significantly different from 0: * *p*-value < 0.05, ** *p*-value < 0.01, ***p-value < 0.001.

## Results

### Per host female mean weight and worm burden

**Figure 5:**
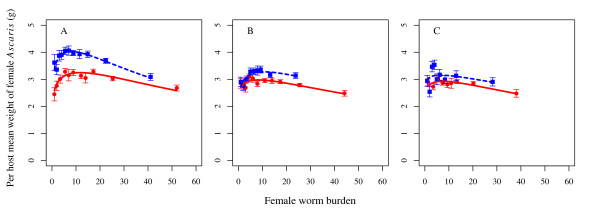
**The best-fit relationship between the per host mean weight of female *Ascaris lumbricoides *and female worm burden**. The best-fit functional relationships (as determined by the LRS, Table 6) between the per host mean weight of female *Ascaris *and the female worm burden in the baseline (A), 1^st ^(B), and 2^nd ^(C) re-infection populations. The solid red line is the best-fit to children (age ≤ 12 years) and the broken blue line to teenagers and adults (age > 12 years). The best-fit function is given by equation (2) and represents a pattern of initial facilitation followed by limitation. Circular and square data points are grouped means for children and teenagers and adults respectively. Error bars represent the standard error of the mean.

Comparisons of nested forms of equation (2) indicated a pattern of facilitation followed by limitation in all populations (Table 6, Figure 5). The LRS and corresponding *p*-values are unambiguous in the baseline and 1^st ^re-infection populations (*p*-value < 0.0001), whereas the facilitation preceding limitation pattern was only marginally preferred over limitation alone in the 2^nd ^re-infection population (*p*-value = 0.046). AIC supported facilitation preceding limitation in all populations. The pattern of density dependence was similar in both age groups, whereas teenagers and adults tended to harbour slightly heavier worms (Figure 5).

**Table 6:** Comparison of models describing density dependence in the mean weight of female *Ascaris lumbricoides*

Population	Model	Model equation	Model description	Test	LRS	*p*-value	AIC
Baseline	A	$\mu_{W}=\alpha_{1}$	no density dependence	-	-	-	4671.33
	B	$\mu_{W}=\frac{\alpha_{1}n}{\left(1+\alpha_{2}n\right)}$	limitation	A vs. B	43.36	<0.0001	4631.97
	C^†^	$\mu_{W}=\frac{\alpha_{1}n^{\left(\alpha_{3}-1\right)}}{\left(1+\alpha_{2}n^{a_{3}}\right)}$	facilitation preceding limitation	B vs. C	38.05	<0.0001	4597.91

1^st ^re-infection	A	$\mu_{W}=\alpha_{1}$	no density dependence	-	-	-	2314.29
	B	$\mu_{W}=\frac{\alpha_{1}n}{\left(1+\alpha_{2}n\right)}$	limitation	A vs. B	18.98	<0.0001	2299.31
	C^†^	$\mu_{W}=\frac{\alpha_{1}n^{\left(\alpha_{3}-1\right)}}{\left(1+\alpha_{2}n^{a_{3}}\right)}$	facilitation preceding limitation	B vs. C	17.99	<0.0001	2285.32

2^nd ^re-infection	A	$\mu_{W}=\alpha_{1}$	no density dependence	-	-	-	1976.29
	B	$\mu_{W}=\frac{\alpha_{1}n}{\left(1+\alpha_{2}n\right)}$	limitation	A vs. B	12.36	0.0021	1967.93
	C^†^	$\mu_{W}=\frac{\alpha_{1}n^{\left(\alpha_{3}-1\right)}}{\left(1+\alpha_{2}n^{a_{3}}\right)}$	facilitation preceding limitation	B vs. C	6.18	0.046	1965.75

Comparison of the goodness-of-fit of special cases of the generalised equation [equation (2)] describing the relationship between the expected value of the per host mean weight of female *Ascaris*, *μ*_*W*_, and the per host female worm burden, *n*, using a likelihood ratio test and AIC; *p*-values were calculated assuming that the LRS follows, under the null hypothesis, a chi-square distribution with 1 d.f. ^† ^denotes the best-fit of each model type in each population.

### Per host female mean weight and net egg output

The LRS and AIC indicated that incorporating the mean weight of female *Ascaris *as a covariate improved the fit of both the NB and ZINB models in all populations (Table 7). The best-fit functional form of the relationship between the per host mean weight of female *Ascaris *and the per host egg output (order of polynomial) varied across populations and with the assumed probability model (Table 8).

**Table 7:** Comparison of models describing the per host net egg output

Population	Model type	Model	Test	LRS	d.f.	*p*-value	AIC
Baseline	Negative binomial	1	-	-	-	-	24679.09
		2	1 vs. 2	50.87	1	<0.0001	24630.21
		3^†^	2 vs. 3	8.35	1	0.0039	24623.86
		4	3 vs. 4	0.02	1	0.89	24625.84
	
	Zero-inflated negative binomial	1I	-	-	-	-	23990.51
		2I	1 vs. 2	26.89	1	<0.0001	23965.62
		3I	2 vs. 3	0.41	1	0.52	23967.20
		4I^†^	3 vs. 4	6.04	1	0.014	23963.17
			2 vs. 4	6.45	2	0.049	

1^st ^re-infection	Negative binomial	1	-	-	-	-	14404.20
		2	1 vs. 2	44.42	1	<0.0001	14361.78
		3	2 vs. 3	4.06	1	0.044	14359.72
		4^†^	3 vs. 4	4.50	1	0.025	14356.72
	
	Zero-inflated negative binomial	1I	-	-	-	-	14043.52
		2I^†^	1 vs. 2	24.52	1	<0.0001	14020.97
		3I	2 vs. 3	0.25	1	0.62	14022.73
		4I	3 vs. 4	0.68	1	0.41	14024.04

2^nd ^re-infection	Negative binomial	1	-	-	-	-	11050.10
		2	1 vs. 2	24.11	1	<0.0001	11027.99
		3^†^	2 vs. 3	5.93	1	0.015	11024.06
		4	3 vs. 4	1.72	1	0.19	11024.33
	
	Zero-inflated negative binomial	1I	-	-	-	-	10715.26
		2I^†^	1 vs. 2	7.24	1	0.0071	10710.02
		3I	2 vs. 3	0.22	1	0.65	10711.81
		4I	3 vs. 4	0.56	1	0.46	10713.26

The goodness-of-fit of all NB and ZINB models fitted to the data assessed by the LRS and AIC. ^† ^denotes the best-fit of each model type in each population. Model equations are given in Table 4.

**Table 8:** Parameter estimates from the null and best-fit statistical models describing per host net egg output

		Negative binomial count component parameters							Bernoulli component parameters			
		
Population	Model	ln(*λ*_1_) (SE)	*β* (SE)	*c* (SE)	*γ*_1_ (SE)	*γ*_2_ (SE)	*γ*_3_ (SE)	*k* (SE)	*δ*_0_ (SE)	*δ*_1_ (SE)	*δ*_2_ (SE)	*δ*_3_ (SE)
Baseline	1 (null)	5.98*** (0.081)	-0.14* (0.068)	0.79*** (0.033)	-	-	-	0.61 (0.020)	-	-	-	-
	3 (best-fit)	6.18*** (0.096)	-0.29*** (0.069)	00.74*** (0.036)	0.20*** (0.026)	-0.046** (0.015)	-	0.63 (0.021)	-	-	-	-
	
	1I (null)	6.25*** (0.066)	-0.20*** (0.053)	0.70*** (0.027)	-	-	-	1.08 (0.034)	0.49* (0.24)	-0.23 (0.28)	-1.02*** (0.14)	-1.78*** (0.18)
	4I (best-fit)	6.26*** (0.076)	-0.28*** (0.055)	0.70*** (0.027)	0.18*** (0.034)	-0.00062 (0.014)	-0.015* (0.0060)	1.10 (0.034)	0.49* (0.24)	-0.23 (0.28)	-1.01*** (0.14)	-1.78*** (0.18)

1^st ^re-infection	1 (null)	5.08*** (0.089)	0.023 (0.078)	1.12*** (0.038)	-	-	-	0.82 (0.036)	-	-	-	-
	4 (best-fit)	5.17*** (0.099)	-0.051 (0.076)	1.11*** (0.039)	0.19** (0.063)	-0.082** (0.031)	0.039* (0.017)	0.86 (0.038)	-	-	-	-
	
	1I (null)	5.31*** (0.081)	0.024 (0.065)	1.02*** (0.035)	-	-	-	1.28 (0.044)	0.91* (0.44)	0.52 (0.40)	-2.00*** (0.33)	-2.65*** (0.37)
	2I (best-fit)	5.28*** (0.080)	-0.027 (0.065)	1.04*** (0.034)	0.18*** (0.037)	-	-	1.31 (0.04)	0.91* (0.44)	0.51 (0.40)	-2.00*** (0.33)	-2.64*** (0.37)

2^nd ^re-infection	1 (null)	5.71*** (0.10)	-0.17 (0.10)	0.81*** (0.047)	-	-	-	0.71 (0.035)	-	-	-	-
	3 (best-fit)	5.77*** (0.11)	-0.23* (0.10)	0.82*** (0.049)	0.27*** (0.047)	-0.078* (0.033)	-	0.74 (0.037)	-	-	-	-
	
	1I (null)	5.95*** (0.087)	-0.14 (0.077)	0.71*** (0.040)	-	-	-	1.23 (0.05)	0.63 (0.41)	1.07** (0.41)	-1.62*** (0.29)	-2.56*** (0.39)
	2I (best-fit)	5.92*** (0.086)	-0.19* (0.079)	0.72*** (0.040)	0.11** (0.042)	-	-	1.24 (0.050)	0.63 (0.41)	1.07** (0.41)	-1.63*** (0.29)	-2.56*** (0.39)

Estimated parameter values from the best-fit NB and ZINB models in each population. Parameters significantly different from 0: * *p*-value < 0.05, ** *p*-value < 0.01, ***p-value < 0.001. Model equations are given in Table 4.

The ZINB model provided a consistently better fit to the data than its non zero-inflated counterpart (Table 7, comparing AIC values) and was able to account for the high proportion of zero counts within the data (Table 9). The vast majority of these zero counts were described in the Bernoulli component of the ZINB model (Table 9); in all populations the per host female worm burden and the per host mean weight of female *Ascaris *were negatively associated with the probability of a zero egg count (Table 8, *p*-value < 0.0001 for both covariates). In the 2^nd ^re-infection population there was also evidence that host age was positively associated with the probability of a zero count (*p*-value = 0.0092 in the best-fit model, Model 2I, Table 8).

**Table 9:** Observed and predicted percentage of zero counts from the NB and ZINB models

Population	Observed percentage zero counts	Predicted percentage zero counts		
		
		NB Model	ZINB Model	
			
			Count component	Bernoulli component
Baseline	7.12%	0.80%	0.039%	7.16%
1^st ^re-infection	5.49%	0.36%	0.033%	5.47%
2^nd ^re-infection	6.66%	0.58%	0.030%	6.64%

The parameter values estimated from the ZINB and NB models are broadly similar (Table 8). The estimated value of the overdispersion parameter *k*, tends to be higher in the ZINB models (indicating reduced overdispersion) since many of the zeros are accounted for in the Bernoulli component of the model (Table 9). It is noteworthy that the estimated values of parameter *c *(the inverse measure of density dependence) tend to be lower in the ZINB models (indicative of more severe density dependence, Table 9).

## Discussion

The major objectives of this study were twofold: Firstly, to determine whether there is any evidence for density-dependent processes affecting the per host mean weight of female *Ascaris lumbricoides*. Secondly, to determine whether per host female mean weight is associated with per host egg output and what, if any, causal impact this has on density-dependent egg production. We have shown that the per host mean weight of female *Ascaris *follows a pattern of initial facilitation followed by limitation with worm burden both at endemic equilibrium (baseline population) and after 6 months re-infection (re-infection populations). An association between the per host mean weight of female *Ascaris *and the per host egg output is demonstrated in the three analysed populations. The functional form of this relationship is different across populations and dependent on the assumed probability model used to estimate the unknown parameters. However, comparing the zero-inflated negative binomial (ZINB) models, which provide a better description of the observed data, we see that at baseline egg output initially rises with increasing per host female mean weight before falling at very high weights, whilst in the re-infection populations, egg output rises monotonically with increasing weight. Despite these findings, per host female mean weight has little discernable causal impact on the well-characterised patterns of density-dependent egg production in *A. lumbricoides *[6,7,70].

The convex pattern of facilitation preceding limitation has been documented in one previous study of the GI-nematode *Heterakis gallinarum *infecting the ring-necked pheasant (*Phasianus colchicus*) [16]. Constraints on female weight may be caused by intra-specific (exploitation) competition for either nutrients or space or by host-mediated effects; such as a non-protective immune response. Limitation of size due to competition for nutrients is unlikely since the total energy requirements of even a heavy *Ascaris *infection is small relative to that of a human host [71] although it may be possible in the severely undernourished. Constraints mediated by the host immune response are also dubitable since teenagers and adults in all populations, who would have a greater immunological experience of *Ascaris *antigens, on average harboured heavier worms. Additionally, negative associations have been reported between various immunological markers and the intensity of *Ascaris *infections [72] and re-infections following chemotherapy [73], suggesting that individuals with heavy worm burdens mount a weaker rather than a stronger immune response. If worm burden relates to the immune response in this manner and the response affects the size of female worms, then the per host mean weight of females would increase with per host worm burden in a facilitative pattern. Immune responses are known to limit worm size in experimental GI infections of rats with *S. ratti *[27,74] and sheep with *T*. *circumcincta *[24,25]. The data presented in this study are not sufficient to distinguish between the various potential causative mechanisms behind the observed density dependence, however, we speculate that the facilitation is immune mediated whereas the limitation is the result of competition for space.

Two previous studies have described a positive relationship between the size of female *A. lumbricoides *and egg production. Sinniah and Subramaniam [14] dissected the uteruses of females expelled from 50 schoolchildren and showed a moderately positive linear relationship. Seo and Chai [15] took a different approach, relating egg output with female length from hosts harbouring a single female or a male and female pair. Their results point to a more parabolic shape to the relationship, with egg output declining in very large (and presumably old) worms. This is in accordance with the results of the present study in the baseline population. Allometric relationships between body size and egg output are a characteristic feature of parasitic nematode infections [75,76], so it is not surprising that similar mechanisms operate in *Ascaris *infections of humans. More interesting, however, is how this association influences the host-parasite interaction and ensuing population dynamics; do host responses limit the size of worms? Are some hosts more efficient than others at doing so? Such processes and heterogeneities are known to occur in model non-human nematode systems [8,27,28].

The degree of density-dependent egg output (described by parameter *c*) remains approximately equal in the null and best-fit models in each of the three populations (regardless of the probability model). This consistency shows that the severity of density dependence is not greatly altered by the effects of female weight. Thus, egg production is limited directly by increasing female worm burden and is not simply an artefact resulting from the density dependence of female mean weight, i.e. the association between egg output and female weight does not cause density-dependent fecundity. It is noteworthy that no statistically significant density-dependent fecundity was detected in the 1^st ^re-infection population (*c *= 1.04, 95% C.I. 0.97–1.11, Model 2I, Table 8). The severity of density-dependent *Ascaris *fecundity is known to be weak in Bangladesh relative to other geographical locations [7], and so its detection is likely to be prone to type II statistical errors.

The relationships between the per host net egg output and the female mean weight varied between the baseline and re-infection populations, with a significant decrease for heavier worms present only at baseline (as indicated by the cubic polynomial providing the best-fit functional relationship, Model 4I). This is congruent with the biological interpretation of this functional form representing a decline in egg production in heavier (inferred older) worms. This would be expected to be unimportant in the populations after six months of re-infection since the life-expectancy of *Ascaris *is estimated to range between 1 and 2 years [1,77].

An important result from this work is the evidence that the per host net egg output tends to be higher in children than adults in the baseline population (*β *= -0.28, *p*-value < 0.0001, Table 8). There is also marginal evidence for this effect in the 2^nd ^re-infection population (*β *= -0.19, *p*-value = 0.015, Table 8). Egg concentration can be negatively associated with the volume of faeces produced resulting in overestimation of egg output in children compared to adults [35]. However, given the unambiguous result in the baseline population it is surprising that the effect is absent and not more statistically significant in the 1^st ^and 2^nd ^re-infection populations respectively. An alternative explanation is that the decreased egg output in adults is due to an acquired immune response. However, to reconcile this with the results from the re-infection populations, the duration of immunological memory should be extremely short and rely on constant exposure to established worms (for transmission models incorporating the effect of immunological memory in helminth infections see [78-80]).

We have shown that a mixture of the negative binomial and Bernoulli distributions (ZINB model) provides a superior description of the distribution of egg output data than a negative binomial (NB model) distribution alone. Similar zero-inflated models have been used frequently in the ecological literature where datasets with many zeros are commonplace (for a review see [81]). In parasitological research, we are aware of only two previous studies that have used zero-inflated models to describe egg output data [45,46]. An added advantage of using a zero-inflated model is the insight which can be gained into the source of zeros (egg counts). Here we show that the probability of a zero count is negatively associated with both the per host female worm burden and the per host female mean weight. These associations suggest that the zero egg counts are false negatives within the data, i.e. failing to detect eggs in truly egg-contaminated faeces. We hypothesise that the greater the total (net) egg production the lower the probability that a sample is taken from a non-contaminated part of the collected faeces. Thus, since total egg production is positively associated both with female worm burden and female mean weight, the probability of sampling from a non-contaminated part of the faeces decreases with increasing female worm burdens and mean weight. This effect will be exacerbated by the highly overdispersed distribution of *A. lumbricoides *eggs in faecal samples [65].

**Figure 6:**
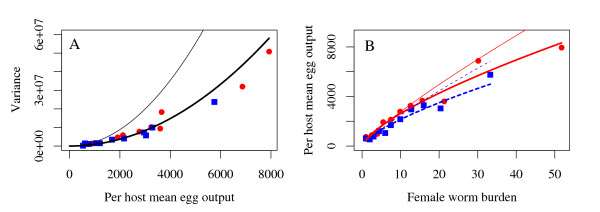
**Comparison of the fit of a log-normal and zero-truncated negative binomial model**. A: The estimated variance-to-mean relationship from the zero-truncated negative binomial model (black thick line) and the log-normal model (black thin line). B: The fitted zero-truncated (thick lines) and log-normal (thin lines) models to data from children (red solid line) and teenagers and adults (blue broken line) in the baseline population. In both figures red circles represent grouped mean data from children and blue squares from teenagers and adults (as defined in Figure 5). Details of the variance-to-mean relationship for the log-normal and zero-truncated negative binomial models are given in additional file 1.

The results presented in Table 9 suggest that a very small fraction of the zeros in the data were generated from the negative binomial count process. If we accept the explanation that the vast majority of zeros are false negatives, it is tempting to remove zero counts *a priori *in order to simplify analyses aimed at detecting epidemiologically significant covariates (i.e. covariates that directly impact upon the release of transmission stages). In taking such an approach one must again choose an appropriate distribution with which to model the now zero truncated data. Two suitable contenders are the log-normal and zero-truncated negative binomial distributions. The advantage of the former is that, via a logarithmic transformation, ordinary least squares estimation procedures can be used. For the latter, numerical maximisation of the appropriate log-likelihood function is required [42,44]. Figure 6 compares the results of fitting equation (5) (in which the mean per host egg output is modelled as being dependent on female worm burden and host age only) to the zero-truncated baseline data using the two approaches. Clearly the log-normal assumption provides an inadequate description of the data due largely to the poor approximation of the variance-to-mean relationship, a key aspect in accurate parameter estimation [40,82] (for details of the variance-to-mean relationship for the log-normal and zero-truncated negative binomial distributions see additional file 1 and [83]). Therefore, although removing zeros from the data may be a reasonable approach, more complex and non-standard statistical models are still required for adequate parameter estimation [42].

## Conclusion

In this study we have demonstrated that the mean weight of female *A. lumbricoides *infecting a cohort of human hosts follows a pattern of facilitation preceding limitation with increasing worm burden. We verify that weight is associated with net egg output but demonstrate that this has little causal impact on patterns of density-dependent egg production. We show that a zero-inflated negative binomial (ZINB) probability distribution is superior to a negative binomial distribution in modelling individual egg output data.

## Competing interests

The authors declare that they have no competing interests.

## Authors' contributions

MW outlined the paper, performed the analyses and wrote the manuscript. AH collected the data and helped in the interpretation of the analyses. M-GB edited the manuscript for intellectual content. RMA, M-GB and AH jointly supervised the work and helped conceive the paper.

## Supplementary Material

Additional file 1 **Probability distribution, variance and expected value for the zero-truncated negative binomial and log-normal distributions. **A file containing the probability distribution, variance and expected value for the zero-truncated negative binomial and log-normal distributions. Click here for file

## References

[B1] Anderson RM, May RM (1992). Infectious Diseases of Humans.

[B2] Anderson RM, May RM (1978). Regulation and stability of host-parasite population interactions I. Regulatory processes. Journal of Animal Ecology.

[B3] Churcher TS (2006). Modelling the Spread of Anthelmintic Resistance. PhD thesis.

[B4] Churcher TS, Basáñez M-G (2008). Density dependence and the spread of anthelmintic resistance. Evolution.

[B5] Churcher TS, Filipe JAN, Basáñez M-G (2006). Density dependence and the control of helminth parasites. Journal of Animal Ecology.

[B6] Anderson RM, May RM (1985). Helminth infections of humans: mathematical models, population dynamics, and control. Advances in Parasitology.

[B7] Hall A, Holland C (2000). Geographical variation in *Ascaris lumbricoides *fecundity and its implications for helminth control. Parasitology Today.

[B8] Stear M, Bairden K, Bishop SC (1997). How hosts control worms. Nature.

[B9] Stear MJ, Bishop SC (1999). The curvilinear relationship between worm length and fecundity of *Teladorsagia circumcincta*. International Journal for Parasitology.

[B10] Richards DT, Lewis JW (2001). Fecundity and egg output by *Toxocara canis *in the red fox, *Vulpes vulpes*. Journal of Helminthology.

[B11] Irvine RJ, Stien A, Dallas JF, Halvorsen O, Langvatn R, Albon SD (2001). Contrasting regulation of fecundity in two abomasal nematodes of Svalbard reindeer (*Rangifer tarandus platyrhynchus*). Parasitology.

[B12] Dezfuli BS, Volponi S, Beltrami I, Poulin R (2002). Intra- and interspecific density-dependent effects on growth in helminth parasites of the cormorant, *Phalacrocorax*. Parasitology.

[B13] Skorping A, Read AF, Keymer AE (1991). Life-history covariation in intestinal nematodes of mammals. Oikos.

[B14] Sinniah B, Subramaniam K (1991). Factors influencing the egg production of *Ascaris lumbricoides*: relationship to weight, length and diameter of worms. Journal of Helminthology.

[B15] Chai JY, Hong ST, Lee SH, Seo BS (1981). Fluctuation of the egg production amounts according to worm burden and length of *Ascaris lumbricoides*. Korean Journal of Parasitology.

[B16] Tompkins DM, Hudson PJ (1999). Regulation of nematode fecundity in the ring-necked pheasant (*Phasianus colchicus*): not just density dependence. Parasitology.

[B17] Michael E, Bundy DAP (1989). Density dependence in establishment, growth and worm fecundity in intestinal helminthiasis: the population biology of *Trichuris muris *(Nematoda) infection in CBA/Ca mice. Parasitology.

[B18] Mello DA (1974). A note on egg production of *Ascaris lumbricoides*. Journal of Parasitology.

[B19] Martin J, Keymer A, Isherwood RJ, Wainwright SM (1983). The prevalence and intensity of *Ascaris lumbricoides *infections in Moslem children from northern Bangladesh. Transactions of the Royal Society of Tropical Medicine and Hygiene.

[B20] Elkins DB, Haswell-Elkins M (1989). The weight/length profiles of *Ascaris lumbricoides *within a human community before mass treatment and following reinfection. Parasitology.

[B21] Holland CV, Crompton DW, Taren DL, Nesheim MC, Sanjur D, Barbeau I, Tucker K (1987). *Ascaris lumbricoides *infection in pre-school children from Chiriqui Province, Panama. Parasitology.

[B22] Monzon RB, Cabrera BD, Cruz AC, Baltazar JC (1990). The "crowding effect" phenomenon in *Ascaris lumbricoides*. Southeast Asian Journal of Tropical Medicine and Public Health.

[B23] Stear MJ, Strain S, Bishop SC (1999). Mechanisms underlying resistance to nematode infection. International Journal for Parasitology.

[B24] Stear MJ, Bishop SC, Doligalska M, Duncan JL, Holmes PH, Irvine J, McCririe L, McKellar QA, Sinski E, Murray M (1995). Regulation of egg production, worm burden, worm length and worm fecundity by host responses in sheep infected with *Ostertagia circumcincta*. Parasite Immunology.

[B25] Stear MJ, Park M, Bishop SC (1996). The key components of resistance to *Ostertagia circumcincta *in lambs. Parasitology Today.

[B26] Pritchard DI, Quinnell RJ, Walsh EA (1995). Immunity in humans to *Necator americanus*: IgE, parasite weight and fecundity. Parasite Immunology.

[B27] Wilkes CP, Thompson FJ, Gardner MP, Paterson S, Viney ME (2004). The effect of the host immune response on the parasitic nematode *Strongyloides ratti*. Parasitology.

[B28] Viney ME, Steer MD, Wilkes CP (2006). The reversibility of constraints on size and fecundity in the parasitic nematode *Strongyloides ratti*. Parasitology.

[B29] Bleay C, Wilkes CP, Paterson S, Viney ME (2007). Density-dependent immune responses against the gastrointestinal nematode *Strongyloides ratti*. International Journal for Parasitology.

[B30] Paterson S, Viney ME (2002). Host immune responses are necessary for density dependence in nematode infections. Parasitology.

[B31] Wilson K, Grenfell BT (1997). Generalized linear modelling for parasitologists. Parasitology Today.

[B32] Paterson S, Lello J (2003). Mixed models: getting the best use of parasitological data. Trends in Parasitology.

[B33] Anderson RM, Schad GA (1985). Hookworm burdens and faecal egg counts: an analysis of the biological basis of variation. Transactions of the Royal Society of Tropical Medicine and Hygiene.

[B34] Croll NA, Anderson RM, Gyorkos TW, Ghadirian E (1982). The population biology and control of *Ascaris lumbricoides *in a rural community in Iran. Transactions of the Royal Society of Tropical Medicine and Hygiene.

[B35] Sinniah B (1982). Daily egg production of *Ascaris lumbricoides*: the distribution of eggs in the faeces and the variability of egg counts. Parasitology.

[B36] Hall A (1981). Quantitative variability of nematode egg counts in faeces: a study among rural Kenyans. Transactions of the Royal Society of Tropical Medicine and Hygiene.

[B37] Guyatt HL, Bundy DAP, Medley GF, Grenfell BT (1990). The relationship between the frequency distribution of *Ascaris lumbricoides *and the prevalence and intensity of infection in human communities. Parasitology.

[B38] Seo B-S, Cho S-Y, Chai J-Y (1979). Egg discharging patterns of *Ascaris lumbricoides *in low worm burden cases. Korean Journal of Parasitology.

[B39] Quinnell RJ, Medley GF, Keymer AE (1990). The regulation of gastrointestinal helminth populations. Philosophical Transactions of the Royal Society of London Series B, Biological Sciences.

[B40] McCullagh P, Nelder JA (1989). Generalized Linear Models.

[B41] Lambert D (1992). Zero-inflated Poisson regression, with an application to defects in manufacturing. Technometrics.

[B42] Hilbe JM (2007). Negative Binomial Regression.

[B43] Ridout M, Demetrio CGB, Hinde J (1998). Models for count data with many zeros. Proceedings of the XIXth International Biometric Conference, Cape Town.

[B44] Welsh AH, Cunningham RB, Donnelly CF, Lindenmayer DB (1996). Modelling the abundance of rare species: statistical models for counts with extra zeros. Ecological Modelling.

[B45] Denwood MJ, Stear MJ, Matthews L, Reid SWJ, Toft N, Innocent GT (2008). The distribution of the pathogenic nematode *Nematodirus battus *in lambs is zero-inflated. Parasitology.

[B46] Nødtvedt A, Dohoo I, Sanchez J, Conboy G, DesCoteaux L, Keefe G, Leslie K, Campbell J (2002). The use of negative binomial modelling in a longitudinal study of gastrointestinal parasite burdens in Canadian dairy cows. Canadian Journal of Veterinary Research.

[B47] Hall A, Anwar KS, Tomkins A (1999). The distribution of *Ascaris lumbricoides *in human hosts: a study of 1765 people in Bangladesh. Trans R Soc Trop Med Hyg.

[B48] Hall A, Anwar KS, Tomkins AM (1992). Intensity of reinfection with *Ascaris lumbricoides *and its implications for parasite control. Lancet.

[B49] Abdi YA, Gustaffson LL, Ericson O, Helgren U (1995). Handbook of Drugs for Tropical Parasitic Infections.

[B50] Keiser J, Utzinger J (2008). Efficacy of current drugs against soil-transmitted helminth infections. Journal of the American Medical Association.

[B51] Basáñez M-G, Remme JH, Alley ES, Bain O, Shelley AJ, Medley GF, Anderson RM (1995). Density-dependent processes in the transmission of human onchocerciasis: relationship between the numbers of microfilariae ingested and successful larval development in the simuliid vector. Parasitology.

[B52] Sinden RE, Dawes EJ, Alavi Y, Waldock J, Finney O, Mendoza J, Butcher GA, Andrews L, Hill AV, Gilbert SC, Basáñez M-G (2007). Progression of *Plasmodium berghei *through *Anopheles stephensi *is density-dependent. PLoS Pathogens.

[B53] Kirkwood BR, Sterne JAC (2003). Essential Medical Statistics.

[B54] Clayton D, Hills M (1993). Statistical Models in Epidemiology.

[B55] Akaike H (1974). New look at statistical model identification. IEEE Transactions on Automatic Control.

[B56] Goldfarb D (1970). A family of variable metric updates derived by variational means. Mathematics of Computation.

[B57] Broyden CG (1970). The convergence of a class of double-rank minimisation algorithms. Journal of the Institute of Mathematics and its Applications.

[B58] Shanno DF (1970). Conditioning of quasi-Newton methods for function minimization. Mathematics of Computation.

[B59] Fletcher R (1970). A new approach to variable metric algorithms. Computer Journal.

[B60] Ihaka R, Gentleman R (1996). R: A language for data analysis and graphics. Journal of Computational and Graphical Statistics.

[B61] Venables WN, Ripley BD (2002). Modern Applied Statistics with S.

[B62] Kleinbaum DG, Kupper LL, Muller KE (1988). Applied Regression Analysis and Other Multivariate Methods.

[B63] Kutner MH, Nachtscheim CJ, Neter J (2004). Applied Linear Regression Models.

[B64] Fox J, Monette G (1992). Generalized collinearity diagnostics. Journal of the American Statistical Association.

[B65] Ye X-P, Donnelly CA, Fu Y-L, Wu Z-X (1997). The non-randomness of the distribution of *Trichuris trichiura *and *Ascaris lumbricoides *eggs in the faeces and the effect of stirring faecal specimens. Tropical Medicine and International Health.

[B66] Mullahy J (1986). Specification and testing of some modified count data models. Journal of Econometrics.

[B67] Dobson AP (2001). An Introduction to Generalized Linear Models.

[B68] Zeileis A, Kleiber C, Jackmon S (2008). Regression models for count data in R. Journal of Statistical Software.

[B69] Greene WH (1994). Accounting for excess zeros and sample selection in Poisson and negative binomial regression models. Economics Working Papers.

[B70] Churcher TS, Ferguson NM, Basáñez M-G (2005). Density dependence and overdispersion in the transmission of helminth parasites. Parasitology.

[B71] Hall A, Hewitt G, Tuffrey V, de Silva N (2008). A review and meta-analysis of the impact of intestinal worms on child growth and nutrition. Maternal and Child Nutrition.

[B72] Turner JD, Faulkner H, Kamgno J, Cormont F, Van Snick J, Else KJ, Grencis RK, Behnke JM, Boussinesq M, Bradley JE (2003). Th2 cytokines are associated with reduced worm burdens in a human intestinal helminth infection. Journal of Infectious Diseases.

[B73] Jackson JA, Turner JD, Rentoul L, Faulkner H, Behnke JM, Hoyle M, Grencis RK, Else KJ, Kamgno J, Boussinesq M, Bradley JE (2004). T helper cell type 2 responsiveness predicts future susceptibility to gastrointestinal nematodes in humans. Journal of Infectious Diseases.

[B74] Moqbel R, McLaren DJ (1980). *Strongyloides ratti*: structural and functional characteristics of normal and immune-damaged worms. Experimental Parasitology.

[B75] Charnov EL (1993). Life History Invariants Some Explorations of Symmetry in Evolutionary Ecology.

[B76] Morand S (1996). Life-history traits in parasitic nematodes: A comparative approach for the search of invariants. Functional Ecology.

[B77] Elkins DB, Haswell-Elkins M, Anderson RM (1986). The epidemiology and control of intestinal helminths in the Pulicat Lake region of Southern India. I. Study design and pre- and post-treatment observations on *Ascaris lumbricoides *infection. Transactions of the Royal Society of Tropical Medicine and Hygiene.

[B78] Woolhouse MEJ (1992). A theoretical framework for the immunoepidemiology of helminth infection. Parasite Immunology.

[B79] Anderson RM, May RM (1985). Herd immunity to helminth infection and implications for parasite control. Nature.

[B80] Berding C, Keymer AE, Murray JD, Slater AF (1986). The population dynamics of acquired immunity to helminth infection. Journal of Theoretical Biology.

[B81] Martin TG, Wintle BA, Rhodes JR, Kuhnert PM, Field SA, Low-Choy SJ, Tyre AJ, Possingham HP (2005). Zero tolerance ecology: improving ecological inference by modelling the source of zero observations. Ecology Letters.

[B82] Warton DI (2005). Many zeros does not mean zero inflation: comparing the goodness-of-fit of parametric models to multivariate abundance data. Environmetrics.

[B83] Lee AH, Wang K, Yau KK, Somerford PJ (2003). Truncated negative binomial mixed regression modelling of ischaemic stroke hospitalizations. Statistics in Medicine.

