# Supplementary material for: Density-dependent effects on the weight of female Ascaris lumbricoides infections of humans and its impact on patterns of egg production
Source: Parasit Vectors. 2009 Feb 10;2:11. doi: 10.1186/1756-3305-2-11 (PMC2672930; doi:10.1186/1756-3305-2-11)
Supplement: Additional file 1 — Probability distribution, variance and expected value for the zero-truncated negative binomial and log-normal distributions. A file containing the probability distribution, variance and expected value for the zero-truncated negative binomial and log-normal distributions. [file 1756-3305-2-11-S1.pdf]

Supplementary material

Probability distribution, variance and expected value for the zero-truncated negative binomial and lognormal distributions

The probability density function (PDF) for the zero-truncated negative binomial distribution is given by the negative binomial distribution rescaled by $1 - P(\Lambda = 0)$,

$$P(\Lambda = \lambda) = \frac{\Gamma(\lambda + k)}{\Gamma(k)\lambda!} \frac{\mu_\Lambda k^k}{(\mu_\Lambda + k)^{\lambda+k}} \frac{1}{1 - \varsigma^k} \qquad \lambda = 1,2 \ldots \tag{S1}$$

where $\varsigma = k/(\mu_\Lambda + k)$. The expected value and variance are [83],

$$E(\Lambda) = \mu_\Lambda/(1 - \varsigma^k) \tag{S2}$$

$$Var(\Lambda) = E(\Lambda)\left(1 + \frac{\mu_\Lambda}{k} + \mu_\Lambda\right) - E(\Lambda)^2 \tag{S3}$$

where μ_Λ is the mean of the original (non-truncated) negative binomial distribution and k is an inverse measure of the degree of overdispersion.

The PDF for the lognormal distribution is,

$$P(\Lambda = \lambda) = \frac{1}{\lambda\sigma_\Lambda\sqrt{2\pi}} \exp\left[-\frac{(\ln(\lambda) - \mu_\Lambda)^2}{2{\sigma_\Lambda}^2}\right] \tag{S4}$$

with expected value and variance,

$$E(\Lambda) = \exp\left(\mu_\Lambda + \frac{{\sigma_\Lambda}^2}{2}\right) \tag{S5}$$

$$Var(\Lambda) = (\exp({\sigma_\Lambda}^2) - 1)\exp(2\mu_\Lambda + {\sigma_\Lambda}^2) \tag{S6}$$

where μ_Λ and ${\sigma_\Lambda}^2$ are the mean and variance of the variable's natural logarithm respectively.
